# Targeting the mTOR Complex by Everolimus in NRAS Mutant Neuroblastoma

**DOI:** 10.1371/journal.pone.0147682

**Published:** 2016-01-28

**Authors:** Michael K. Kiessling, Alessandra Curioni-Fontecedro, Panagiotis Samaras, Silvia Lang, Michael Scharl, Adriano Aguzzi, Derek A. Oldrige, John M. Maris, Gerhard Rogler

**Affiliations:** 1 Department of Gastroenterology and Hepatology, UniversityHospital Zurich, Zurich, Switzerland; 2 Department of Oncology, University Hospital Zurich, Zurich, Switzerland; 3 Institute of Neuropathology, University Hospital Zurich, Zurich, Switzerland; 4 Children's Hospital of Philadelphia, Perelman School of Medicine at the University of Pennsylvania, Philadelphia, United States of America; University of Navarra, SPAIN

## Abstract

High-risk neuroblastoma remains lethal in about 50% of patients despite multimodal treatment. Recent attempts to identify molecular targets for specific therapies have shown that Neuroblastoma RAS (NRAS) is significantly mutated in a small number of patients. However, few inhibitors for the potential treatment for NRAS mutant neuroblastoma have been investigated so far. In this *in-vitro* study, we show that MEK inhibitors AZD6244, MEK162 and PD0325901 block cell growth in NRAS mutant neuroblastoma cell lines but not in NRAS wild-type cell lines. Several studies show that mutant NRAS leads to PI3K pathway activation and combined inhibitors of PI3K/mTOR effectively block cell growth. However, we observed the combination of MEK inhibitors with PI3K or AKT inhibitors did not show synergestic effects on cell growth. Thus, we tested single mTOR inhibitors Everolimus and AZD8055. Interestingly, Everolimus and AZD8055 alone were sufficient to block cell growth in NRAS mutant cell lines but not in wild-type cell lines. We found that Everolimus alone induced apoptosis in NRAS mutant neuroblastoma. Furthermore, the combination of mTOR and MEK inhibitors resulted in synergistic growth inhibition. Taken together, our results show that NRAS mutant neuroblastoma can be targeted by clinically available Everolimus alone or in combination with MEK inhibitors which could impact future clinical studies.

## Introduction

Neuroblastoma is a developmental tumor of early childhood arising from the neural crest [1, 2]. Neuroblastomas show biologic heterogeneity spanning a wide range of clinical behaviors from spontaneous regressions to lethal outcome. High-risk patients account for 50% of all new neuroblastoma diagnosis and cause about 13% of all pediatric cancer mortality despite multimodal treatment [1]. To improve therapy by identifying novel targets, four studies performing genome sequencing of 36–240 patients detected point mutations and structural alterations in ARID1A/B, PTPN11, MYCN, ALK and NRAS [3–5]. Anaplastic lymphoma kinase (ALK) has been studied as a putative drug target. ALK is mutated in about 8% of primary neuroblastomas and can be blocked by ALK inhibitors such as Crizotinib which reduce cell growth and induce apoptosis in cell lines [6, 7]. Two NRAS and one HRAS mutation were described in two of the genomic landscape studies of neuroblastoma [4, 5]. NRAS mutations are found in various cancers including melanoma (20–25%), lung cancer (1%), acute myeloid leukemia (10%) and cutaneous T-cell lymphoma patients (4%) [8–10]. Mutations of NRAS are found at typical hotspots including codon 12, 13 and 61 which results in G12C/S, G13R/V and Q61R/L mutations. These mutations block GTPase activity and lock the RAS isoforms in continuous activation in which they signal to downstream effectors such as MEK and ERK [11]. Direct targeting of mutant NRAS by farnesylation inhibitors have failed but blocking downstream MEK kinase by MEK kinase inhibitors was successful in a preclinical setting. Different MEK inhibitors specifically block cell growth and induce apoptosis in melanoma, neuroblastoma, lung cancer and T-cell lymphoma cell lines [9, 12, 13]. More important, targeting NRAS by MEK inhibitors MEK162 (Binimetinib) was shown to be efficient in a phase II clinical trial with 30 melanoma patients [14]. Partial responses were observed in 20% of treated patients while 43% were presenting with stable disease [14]. Several studies could show that mutant NRAS activates the PI3K/mechanistic target of rapamycin (mTOR)-signaling cascade in melanoma and lung cancer [13, 15]. Further, combined inhibition of MEK and PI3K pathways was synergistic [13, 15]. However, these studies used combined PI3K/mTOR inhibitors currently in preclinical and early clinical evaluation [13, 15]. It is not known whether inhibition of both targets is required or whether inhibition of PI3K or mTOR alone would suffice to impact tumor growth and survival.

Here, we investigated the druggability of NRAS in neuroblastoma. We show that NRAS can be targeted by MEK inhibitors AZD6244 (Selumetinib) and MEK162 whereas wild-type cells are refractory to this treatment. We observed that single PI3K inhibitors were ineffective in blocking PI3K signaling and also cell growth. However, the use of single mTOR inhibitors Everolimus and AZD8055 resulted in a significant reduction of cell growth exclusively in NRAS mutant cell lines. More importantly, Everolimus alone was sufficient to induce apoptosis in NRAS mutant neuroblastoma cell lines but not in wild-type cell lines. Moreover, combination of mTOR and MEK inhibitors block cell growth synergistically. These findings open new perspectives in the treatment of high-risk and relapsed neuroblastoma.

## Methods

### Patient Samples

Patient samples are derived from high-risk neuroblastoma patients as described in [4] as part of the NCI funded TARGET project (https://ocg.cancer.gov/programs/target). The Children's Hospital of Philadelphia Institutional Review Board approved this study and was responsible for oversight of this study. Flash-frozen tumor samples were analyzed for percent tumor content by histopathology, and samples with <75% tumor content were excluded.

### Chemicals

AZD6244, MEK162, BKM120 and PD0325901 were purchased from Selleck Chemical. Everolimus, AZD8055, GSK690693, MK2206 and BKM120 (as a control) were purchased from MedChemExpress. All inhibitors were solubilized in dimethyl sulfoxide (DMSO) at stock concentrations of 1mM.

### Cell Culture

CHP-212 and SK-N-AS were purchased from ATCC. NGP and CHP-134 were purchased from DSMZ (Deutsche Sammlung von Mikroorganismen und Zellkulturen, Germany). SH-SY5Y was provided by Prof. A. Aguzzi, Institute of Neuropathology, Zurich. CHP-212 cells were cultured in DMEM and F12 medium (1:1) supplemented with 10% fetal calf serum (FCS) and 1mM L-glutamine. SK-N-AS cells were cultured in DMEM supplemented with 0.1mM non-essential amino acids and 1mM L-glutamine. NGP cells were cultured in high-glucose DMEM (4.5 g/L glucose) supplemented with 10% FCS. SH-SY5Y cells were cultured in DMEM supplemented with 10% FCS. All cell lines were purchased within less than one year and regularly tested for mycoplasma contamination.

### siRNA Transfection and Knock-Down

CHP-212, SK-N-AS, SH-SY5Y, CHP-134 and NGP were transfected with either control or two different siRNAs against NRAS (Dharmacon, A-003919-13-0005, A-003919-14-0005). Accell siRNA was purchased from Dharmacon and used according to manufactor’s protocol. In brief, siRNAs were diluted in Accell siRNA Delivery Media from Dharmacon to a final concentration of 1μM. Knockdown efficiency was observed after 96 hours.

### Western Blot Analysis

Western Blots were performed as described previously [9]. In brief, 0.5x10^6^ cells were lysed for 30 minutes in ice-cold MPERM buffer supplemented with 25mM NaF, 1mM dithiothreitol, and complete protease inhibitor cocktail from Roche Diagnostics. Cell debris was removed by centrifugation and then, proteins were blotted onto a nitrocellulose membrane (GE Healthcare) followed by blocking with 5% bovine serum albumin in phosphate-buffered saline/Tween (0.05% Tween-20 in phosphate-buffered saline). The following antibodies were used: anti-phospho-ERK (P-p44/p42 (Tyr202/204, #9101, Cell Signaling Technology), anti-ERK (p44/p42, # 4695, Cell Signaling Technology), anti-phospho Akt (Ser473, # 4085, Cell Signaling Technology), anti-panAKT (# 9272, Cell Signaling Technology), anti-phospho MEK (Ser298, #9128, Cell Signaling Technology), anti-MEK (# 8727, Cell Signaling Technology), anti-phospho mTOR (Ser2448, #2971, Cell Signaling Technology), anti-mTOR (# 2972, Cell Signaling Technology), anti-phospho S6 (Ser235/236, Cell Signaling Technology), anti-S6 (#2317, Cell Signaling Technology), anti-NRAS rabbit (# ab97488, Abcam) and anti—tubulin (Sigma-Aldrich).

### PCR and Sequencing of Cell Lines

Identification of mutations was performed as described previously [9]. Total cellular RNA was isolated using the Qiagen RNA Purification Kit (Qiagen). 1μg of RNA was reverse transcribed with a reverse transcription-PCR kit (Applied Biosystems); 5 μL of cDNA was used for a PCR of 50 μL volume. The following primers were used: NRAS forward, 5’-ggggtctccaacatttttcc-3’; NRAS reverse, 5’-cccagggcagaaaaataaca-3’. PCR was performed, and 30 μL of PCR product was sent for sequencing to Microsynth, Switzerland. For sequencing, the same primers were used as for PCR. Mutations were verified in Cancer Cell Line Encyclopedia or COSMIC database [16].

### Apoptosis Assays

Cell lines were treated with indicated concentrations of inhibitors and apoptosis was measured after 48 hours and 72 hours. Apoptosis was assessed by AnnexinV—APC (Enzo Lifescience) and propidium iodide (PI) (Sigma-Aldrich) by FACS. Both reagents were diluted to a final concentration of 1.5%. Specific cell death was calculated by the following equation: specific cell death % = (% experimental cell death—% spontaneous cell death)/(100%—% spontaneous cell death) x 100 [17].

### Cell Growth and Viability Assays

Cell growth was measured with the Cell-Titer-Glo Reagent (Promega) according to manufacturer’s instructions. Cells were plated in 96-well plates at a density of 500–2500 cells per well. The next day, drugs were added at indicated concentrations and cell growth was measured 96 hours later. Proliferation was made with a 96-well plate luminometer/plate reader (Synergy 2, Biotek). Data shown are calculated as relative values for a given drug concentration compared to non-treated cells. All experimental points were set up in duplicate replicates and were conducted at least 3 independent times. IC50 were calculated with GraphPad Primsm. Combination index (CI) values with CalcuSyn Software (Biosoft) according to Chou [18]. CI values less than one were considered synergistic.

## Results

### Characteristics of Patients with Neuroblastoma that Harbor NRAS Mutations

Within the TARGET consortium whole genome or whole exome sequencing of newly diagnosed matched neuroblastoma tumor and blood-derived DNAs was performed [4, 19]. We found 3 NRAS-mutated cases in 299 predominantly high-risk patients with whole genome or whole exome sequencing (Table 1). One additional NRAS-mutated case was identified in additional 500 patients who are of low-, intermediate- and high-risk neuroblastoma. In total, we identified 4 NRAS-mutant cases among 799 tested (0.5%) and, 3 out of 4 mutations occurred in high-risk patients (Table 1). One of the tumors had a NRAS G13R while 3 harbored a NRAS G61K mutation (Table 1). Clinical characteristics of patients with NRAS mutations are summarized in Table 1.

**Table 1:** Characteristics of individual patients with NRAS-mutant tumors.

	RAS mutation	Gender	INSS Stage	COG risk group	MYCN status	OS (Days)	treatment OS
**Patient 1**	**NRAS (G13R)**	Female	Stage 4	High	Not Amplified	647	Dead
**Patient 2**	**NRAS (Q61K)**	Female	Stage 4	High	Amplified	765	Alive
**Patient 3**	**NRAS (Q61K)**	Female	Stage 4	Intermediate	Not Amplified	480	Dead
**Patient 4**	**NRAS (Q61K)**	Female	Stage 4	High	Not Amplified	521	Alive

### MEK Inhibitors Block Cell Growth in NRAS Mutated Neuroblastoma Cancer Cell Lines

Analysis of the NRAS gene in our panel of neuroblastoma cell lines revealed two NRAS mutations at position Q61K in the CHP-212 and SK-N-AS cell line, which was also the most common mutation in the primary tumors (Tables 1 and 2).

**Table 2:** NRAS und ALK mutations in selected neuroblastoma cell lines. Cell lines were sequenced for NRAS mutations. Results were evaluated by Genetic Analysis Technology Consortium sequence viewer.

	NRAS	ALK
CHP-212	Q61K	-
SK-N-AS	Q61K	-
SH-SY5Y	-	F1174L
CHP-134	-	-
NGP	-	-

**Fig 1:**
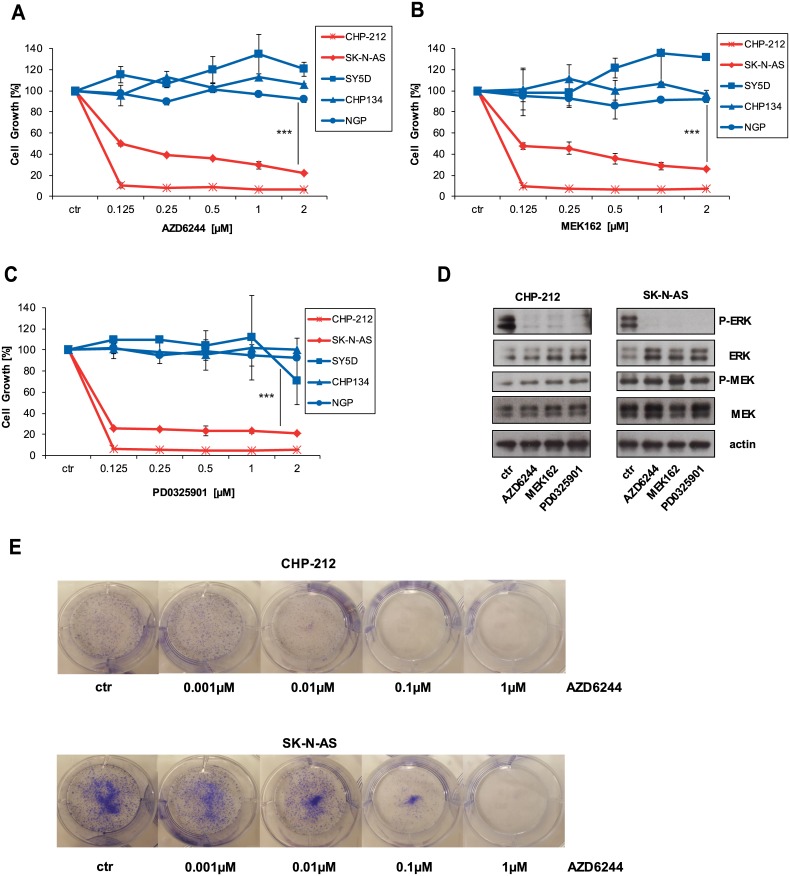
MEK inhibition blocks cell growth in NRAS mutant cells. A-C) All indicated cell lines were kept under equal conditions. Cell lines were left untreated or treated with indicated concentrations of the 3 MEK inhibitors AZD6244, MEK162 and PD0325901 for 96 hours. Then, cell growth was measured by ATP changes by Cell Titer Glo according to the manufacturer’s instructions. IC50 were calculated with GraphPad Prims and statistical difference calculated with t-test. *** p < 0.0001. D) All cell lines were kept under equal conditions, then treated with 500nM of the MEK inhibitors AZD6244, MEK162 and PD0325901 for 1 hour and next lysed and subjected to Western blot. Phosphorylation levels of ERK and MEK were detected by specific anti-phospho antibodies. Loading was verified by specific antibodies to total ERK, MEK and anti—tubulin. E) Crystal violet stain of CHP-212 and SK-N-AS cells from a clonogenic assay after 10 d treatment with DMSO (ctr), 0.001μM, 0.01 μM, 0.1μM and 1μM MEK162.

The identified mutations were found to be heterozygous (Table 2). These mutations are well-known to lead to oncogenic activation of NRAS [11]. To investigate the effect of MEK inhibition, we applied three different MEK inhibitors; two are currently in clinical development [14, 20]. We observed dose-dependent growth inhibition of the NRAS mutant neuroblastoma cell lines CHP-212 and SK-N-AS treated with MEK inhibitors AZD6244, MEK162 and PD0301925 (Fig 1A–1C). These results are in agreement with previously published data showing that NRAS mutations sensitize towards MEK inhibitors [9, 12, 13, 15]. NRAS wild-type neuroblastoma cell lines SH-SY5Y, CHP-134 and NGP were refractory to MEK inhibitors even at high micromolar concentrations. The calculated IC50 values for AZD6244 for CHP-212 and SK-N-AS were 0.8nM and 418nM respectively (S1B Fig). Of note, these are clinically achievable plasma concentrations since maximal plasma concentrations of AZD6244 and MEK162 were at 1.75 μM and 1.13 μM, respectively [21, 22]. In line with these observations, inhibition of MEK blocks downstream ERK phosphorylation in NRAS mutant cell lines (Fig 1D). MEK phosphorylation itself is not blocked by MEK inhibitors but rather increased as shown previously [9, 23]. Finally, MEK162 suppressed clonogenic growth in NRAS mutant cell lines CHP-212 and SK-N-AS for 10 days (Fig 1E). Taken together, these data show that NRAS Q61K mutations in neuroblastoma result in hyperactivation of the RAS/RAF/MEK/ERK pathway and sensitize to MEK inhibitors.

### Mutant NRAS Is Indispensable for Survival of NRAS Mutant Neuroblastoma

The effect of NRAS knock-down was studied to investigate whether NRAS mutations are required for survival of mutant cell lines. We performed a specific NRAS knock-down with two different siRNAs. The knock-down efficiency of the siRNAs was comparable in all cell lines (S1C Fig). Interestingly, the NRAS Q61K neuroblastoma cell lines SK-N-AS and CHP-212 showed a significant decrease in cell growth after siRNA mediated NRAS knock-down, compared to cells transfected with non-targeting control siRNA (S1B Fig). We conclude that NRAS activity is required for viability of NRAS mutant neuroblastoma. In addition, we found that MEK inhibitor MEK162 might induce cell death in NRAS mutant cell lines CHP-212 and to a lower extend in SK-N-AS (S2A Fig).

### NRAS Mutant Neuroblastoma Is Insensitive to PI3K Inhibition

**Fig 2:**
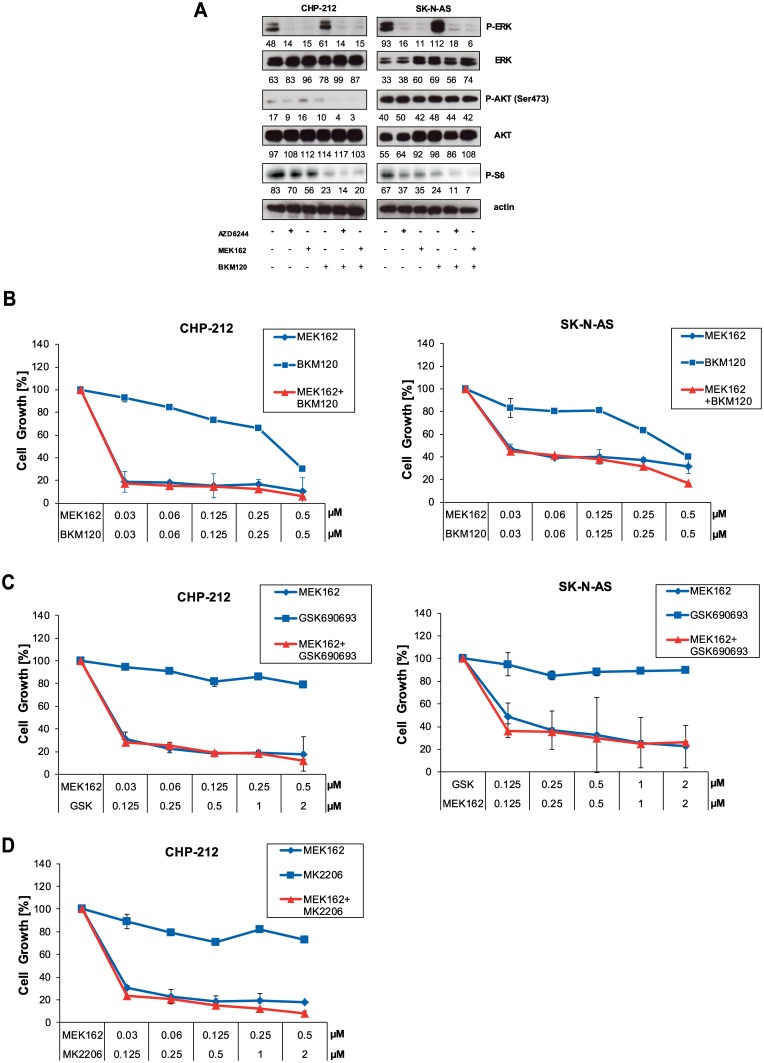
Blocking the PI3K pathway does not influence cell growth or signaling. A) CHP-212 and SK-N-AS cells were treated with 500nM of the MEK inhibitor MEK162 or the PI3K inhibitor BKM120 for 1 hour and next lysed and subjected to Western blot. Phosphorylation levels of AKT, ERK and S6 were detected by specific anti-phospho antibodies. Loading was verified by specific antibodies to total AKT, ERK and anti—tubulin. B) CHP-212 and SK-N-AS cells were treated with 500nM of MEK162 or BKM120 or combinations thereof as indicated for 96h. Then, cell growth was assessed by Cell titer Glo. C) Same as B) but the AKT1/2/3 inhibitor GSK690693 was used instead of BKM120. D) Same as C) but the AKT1/2/3 inhibitor MK2206 was used instead of GSK690693.

Recently published studies showed that mutant NRAS melanoma and neuroblastoma cells are highly sensitive to the combination of MEK and combined PI3K/mTOR inhibitors which are in preclinical or early clinical development [13, 15]. However, it is unclear whether inhibition of either PI3K or mTOR alone is sufficient. Thus, we intended to examine the growth inhibitory effect of single PI3K inhibitors or in combination with MEK inhibitors. However, we found that activation of AKT measured by phosphorylation levels of AKT Ser473 did not change remarkably upon PI3K inhibitor treatment with BKM120 (Buparlisib) (Fig 2A). Thus, we further investigated phosphorylation levels at AKT Thr308. However, background phosphorylation of Thr308 was barley detected in CHP-212 and SK-N-AS cell lines (S3A Fig). To assure that the BKM120 would be generally functional in this setting, we used the multiple myeloma cell line L-363 as a positive control. In previous screening experiments in our lab, L-363 was one of the very few cell lines that showed a relevant IC50 towards BKM120 (data not shown). Indeed, BKM120 and another PI3K inhibitor GDC0032 effectively blocked AKT phosphorylation at Ser473 in L-363 (S3B and S3C Fig) proving that BKM120 is able to block the PI3K pathway in certain cell lines. Regarding the NRAS mutant neuroblastoma, this might implicate that phosphorylation of AKT is independent of upstream PI3K activity in these cell lines and that mutant NRAS does not signal via the PI3K/AKT axis. These observations were supported by the finding of cell growth experiments. Combination of BKM120 with MEK inhibitor MEK162 did not enhance cell growth inhibition compared to MEK inhibitor treatment alone in both cell lines (Fig 2B). To further support these data, we used the AKT1/2/3 inhibitors GSK690693 and MK2206. GSK690693 alone or in combination with MEK162 had no effect on cell growth (Fig 2C). We obtained a comparable result for MK2206 for the CHP-212 cell line (Fig 2D). Other reports have shown that combined inhibition by PI3K/mTOR inhibitors block cell growth [13]. Our data suggest that the PI3K/AKT pathway is, at least in these model systems, not relevant for survival and cell growth of NRAS mutant neuroblastoma cells.

### mTOR Inhibitor Everolimus in Single Use Reduces Cell Growth and Leads to Apoptosis in NRAS Mutant Neuroblastoma Cell Lines

**Fig 3:**
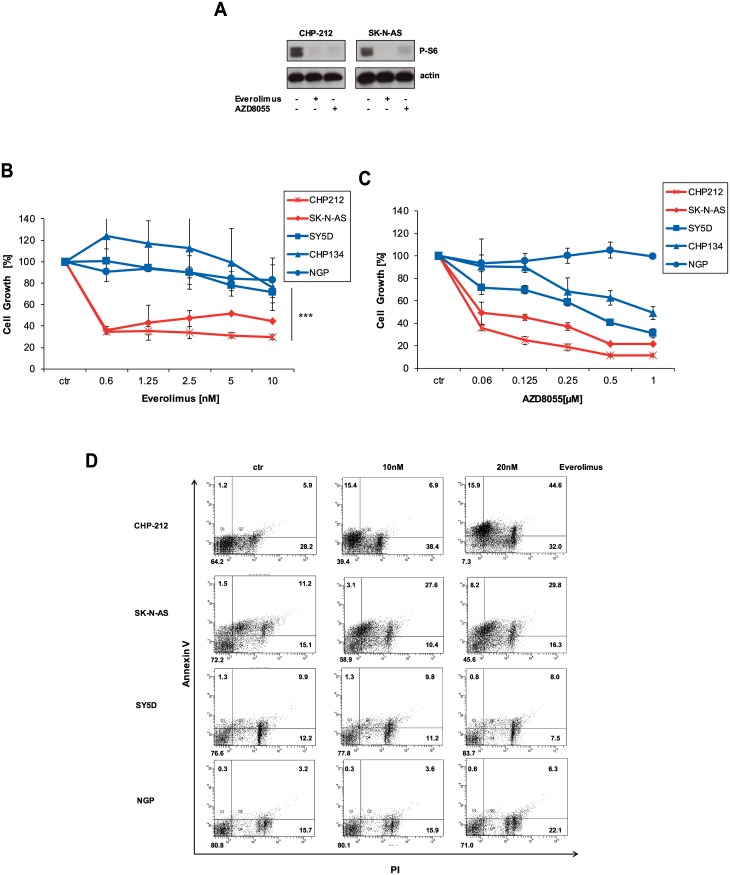
Inhibition of mTOR reduces cell growth in NRAS mutant cell lines. A) CHP-212 and SK-N-AS cells were treated with 5nM of the mTOR inhibitors Everolimus or 250nM AZD8055 for 1 hour. Then, cells were lysed and analysed by Western blot. B) All indicated cell lines were left untreated or treated with indicated concentrations of Everolimus for 96 hours. Next, cell growth was measured by Cell Titer Glo according to the manufacturer’s instructions C) Same as B) but the mTOR inhibitor AZD8055 was used instead. D) NRAS mutant and wild-type cell lines were incubated with indicated concentrations of Everolimus for 72 hours. Then, apoptosis was determined by Annexin V and PI staining.

**Fig 4:**
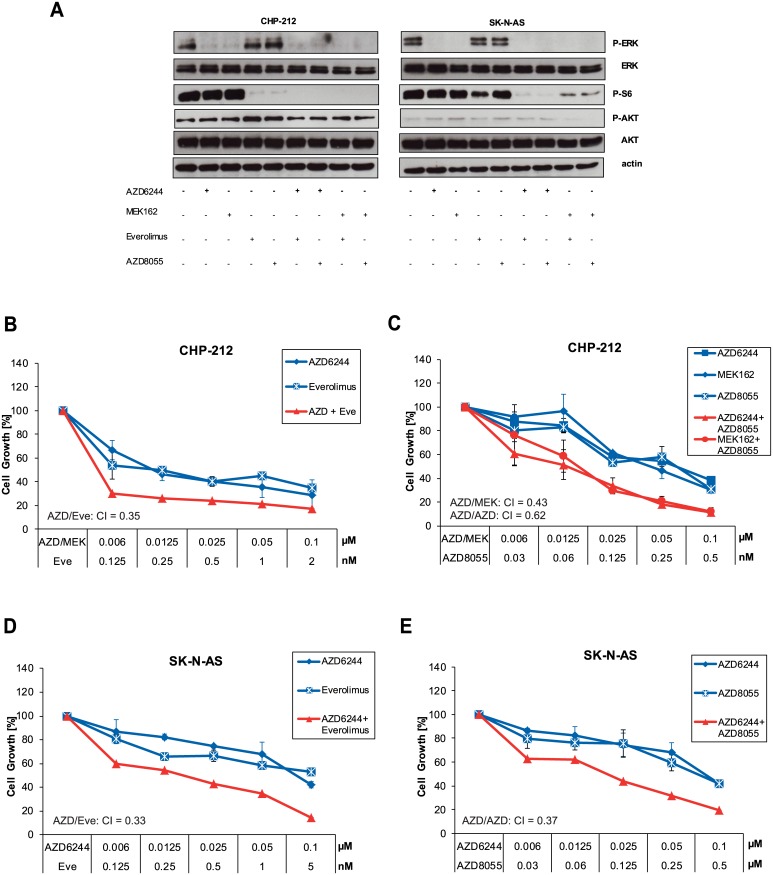
Combined blockage of mTOR and MEK pathways reduces cell growth synergistically. A) CHP-212 and SK-N-AS cells were treated with indicated concentrations of AZD6244, MEK162, Everolimus or AZD8055 or combinations thereof as indicated for 1 hour. Then, cells were lysed and analysed by Western blot. Phosphorylation levels of AKT, ERK and S6 were detected by specific anti-phospho antibodies. Loading was verified by specific antibodies to total AKT, ERK and anti—tubulin. B) CHP-212 cells were treated with indicated concentrations of AZD6244, MEK162 or Everolimus or combinations thereof for 96h. Then, cell growth was measured by Cell Titer Glo. Combination index (CI) values with CalcuSyn Software (Biosoft). C) Same as B) but AZD8055 was used instead of Everolimus. D) SK-N-AS cells were treated with indicated concentrations of AZD6244 or Everolimus or combinations thereof for 96h. Then, cell growth was measured by Cell Titer Glo. E) Same as D) but AZD8055 was used instead of Everolimus.

Targeting of the PI3K/AKT pathway seemed not to have some effect on NRAS mutant neuroblastoma cells. Since combined inhibitors of PI3K/mTOR showed activity in previous studies, we hypothesized that inhibition of the down-stream mTOR complex could be sufficient. We chose the FDA-approved mTOR inhibitor Everolimus which would be readily available for further clinical evaluation. As a control, the mTOR kinase inhibitor AZD8055, which inhibits both mTORC1 and mTORC2 complexes, was used [24]. Both inhibitors showed equally capability in blocking mTOR assessed by phosphorylation levels of downstream S6 kinase (Fig 3A). Strikingly, inhibition of the mTOR pathway alone resulted in a strong blockage of cell growth in NRAS mutant neuroblastoma cancer cell lines compared to wild-type (Fig 4B). IC50 values for CHP-212 and SK-N-AS were both <1nM (Fig 3B). Maximal plasma concentrations for Everolimus in patients with pediatric tumors were reported to be between 16.7nM and 97.8nM depending on dosing applied [25]. Mean plasma concentrations over 24 hours for Everolimus ranged between 6-14nM [25]. This indicated that IC50 values for both NRAS mutant neuroblastoma cell lines were at a sensitive level for plasma concentrations of Everolimus. In addition, the mTOR1/2 inhibitor AZD8055 showed a similar outcome (Fig 3C). AZD8055 blocked cell growth also in wild-type cell lines, though to a lesser extent than in NRAS mutant cell lines (Fig 3C). This might indicate a certain degree of dependency on mTORC2 signaling in wild-type cell lines. Next, we evaluated whether inhibition of cell growth by Everolimus also inducted apoptosis. Indeed, Everolimus caused significant apoptosis exclusively in NRAS mutant cell lines but not in NRAS wild-type cell lines (Fig 3D). In summary, inhibition of mTOR complex alone by Everolimus reduced cell growth and induced apoptosis in NRAS mutant neuroblastoma.

### Combined Inhibition of MEK and mTOR Synergistically Reduces Cell Growth in NRAS Mutant Neuroblastoma Cell Lines

Encouraged by these findings, we aimed to investigate how the combination of mTOR inhibitors and MEK inhibitors would affect cell growth of NRAS mutant neuroblastoma cell lines. If used together, the combination of Everolimus and AZD6244 / MEK162 caused a stronger inhibition of S6 kinase than monotherapy as detected by Western Blot (Fig 4A). The combination of Everolimus and AZD6244 / MEK162 also resulted in a stronger blockade of cell growth in NRAS mutant cells than monotherapy (Fig 4B and 4C). To further strengthen our results, we investigated the effect at different timepoints. We found that synergistic activity can be observed after 3 or 4 days whereas day 2 might to early to observe relevant toxicity (S4A–S4C Fig). Notably, concentrations used for combined inhibition had very low nanomolar concentrations for both Everolimus and AZD6244 or MEK162 (Fig 4B and 4C). Importantly, the combination of Everolimus and MEK inhibitors was found to have significant synergistical effect according to Chou [18]. Synergy is defined if the combination index (CI) is below 1 [18]. We found the CI values to be between 0.29 and 0.43 which indicates strong synergy (Fig 4B and 4C). Also the combination of AZD8055 and MEK was synergistic. AZD8055 blocked downstream phosphorylation of S6 kinase alone (Fig 4A). Yet, phosphorylation of S6 kinase and cell growth was further blocked when AZD8055 was used in combination with MEK inhibitors (Fig 4A, 4D and 4E). To summarize, these data show that combination of mTOR and MEK inhibitors synergistically inhibit downstream signaling and cell growth of NRAS mutant cell lines.

## Discussion

NRAS mutations are found in various malignancies including melanoma (20%), adenocarcinoma of the lung (1%), neuroblastoma (0.83%) and cutaneous T-cell lymphoma (4%). We and others have shown that NRAS mutations sensitize towards inhibition of MEK in cutaneous T-cell lymphoma, lung cancer and neuroblastoma cell lines [9, 12, 13, 15]. These findings are now further supported by demonstrating that knock-down of mutant NRAS causes growth inhibition exclusively in NRAS mutant neuroblastoma cell lines (S1 Fig). Many studies explore the potential use of dual inhibition of MAPK and PI3K/AKT/mTOR which results in growth inhibition in NRAS mutant melanoma and neuroblastoma [13, 15]. However, combined inhibitors targeting both PI3K and mTOR were used in these studies. We found that single inhibition of PI3K or AKT alone had no effect on cell growth of NRAS mutant neuroblastoma which is concordant with the results from Vujic et al. (Fig 2) [15]. We observed that the PI3K or AKT inhibitors alone or in combination with MEK inhibitors did not alter cell growth (Fig 2). However, we found that BKM120 alone or in combination with MEK inhibitors does block S6 phosphorylation downstream independent of its activity on AKT phosphorylation (Fig 2). Further, this activity couldn´t be correlated to effects on cell growth. We thus assume that BKM120 might have unspecific activity on PI3K/AKT or that the activity has to be correlated to other currently unknown biomarkers than AKT and S6 phosphorylation. Recently, it was shown that the combination of MEK inhibitor AZD6244 and AKT inhibitor MK2206 are effective in KRAS mutated cancers [26]. The combination treatment of AKT and MEK inhibitiors was significantly synergistic compared to mono therapy [26]. Moreover, this early trial reported an impressive response rate of AKT and MEK combination in KRAS mutant non-small cell lung cancer (NSCLC) patients [26]. However, we could not observe a synergism of AKT inhibitor MK2206 and MEK inhibitors AZD6244 and MEK162 in NRAS mutant neuroblastoma cell lines (data not shown). This might be explained by different downstream signaling of the different RAS family members. For instance, BRAF and NRAS mutations can be efficiently targeted by MEK inhibitors alone both in cell lines and in patients, resulting in significant response rates [27–29]. However, KRAS mutations alone are not a strong indicator for response to MEK inhibitors demonstrated by a study that shows that roughly one half of cell lines with KRAS mutations responded to AZD6244 whereas the other half did not [30]. Therefore, it has been speculated whether additional cellular pathways are involved in mediating the viability of KRAS-transformed cells which supports the concept of synthetic lethality [31].

In our study, we demonstrate that inhibition of the mTOR pathway by Everolimus alone is sufficient to block cell growth in NRAS mutant neuroblastoma cells compared to wild type cells (Fig 3). More importantly, Everolimus induced apoptosis significantly in NRAS mutant neuroblastoma CHP-212 cells (Fig 3). This is an intriguing finding since Everolimus is currently indicated and available for the treatment of renal cell cancer and pancreatic neuroendocrine tumors and could be of value for carefully selected patients with NRAS mutant neuroblastomas. Since blocking mTOR alone seemed to generate a similar outcome in cell growth as MEK inhibitors, we speculated whether combination of both drugs would be able to further potentiate inhibition. Interestingly, combination of MEK and mTOR inhibitors is synergistic in NRAS mutant neuroblastoma cell lines which were observed for both cell lines (Fig 4). Whether combinations of MEK and mTOR or even MEK and combined AKT/mTOR inhibitors can be used in clinical setting regarding site effects is currently investigated. Phase I trials reported good tolerability for the combination of the MEK inhibitor pimasertib and the PI3K/mTOR inhibitor SAR245409 in 53 patients [32]. Currently, it is unclear how mutant NRAS signals to the mTOR complex without involving upstream PI3K and AKT. This merits further investigation to maybe improve combined targeting of both pathways or to identify other involved molecules that could be directly targeted.

In conclusion, we show that mutant NRAS neuroblastoma can be targeted by single MEK and mTOR inhibitors or their combinations. Especially Everolimus as currently available drug could be an alternative option for NRAS mutated neuroblastoma patients being refractory to other treatments.

## Supporting Information

S1 Fig Mutations in neuroblastoma cell lines and Knock-down of mutant NRAS leads to inhibition of cell growth. A) PCR obtained from cDNA of respective cell lines were sequenced by Sanger sequencing for the complete NRAS coding region. B) Cell lines were incubated with indicated concentrations of MEK162 and IC50 values were calculated with GraphPad Prism. C) Accell siRNA against NRAS (siNRAS1 and siNRAS2) was transfected in NRAS mutant cancer cell lines according to manufactor´s instruction. Then, 96h later cells were lysed and lysates subjected to Western blot. Efficiency of knock-down was measured by anti-NRAS antibodies. Equal loading was controlled by anti-tubulin antibodies. D) Accell siRNA against NRAS (siNRAS1 and siNRAS2) was transfected in NRAS mutant cancer cell lines according to manufactor´s instruction. 96 hours after transfection cell growth was assessed by Cell titer Glo. P values were calculated with t-test and * defined as p<0.05.
(EPS)

S2 Fig MEK162 induces cell death in NRAS mutant cell lines. A) NRAS mutant and NRAS wild-type cell lines were incubated with indicated concentrations of MEK inhibitors MEK162 for 72 hours. Then, cell death was determined by Annexin V and PI staining.
(EPS)

S3 Fig BKM120 does not affect AKT phosphorylation in neuroblastoma but in sensitive lymphoma cell lines. A) CHP-212, SK-N-AS and BT-474 (used as planned positive control) cells were treated with 0.5μM and 1μM of BKM120 for 3 hours. Then, cells were lysed and analysed by Western blot. B) L-363 was used a positive control to investigate whether BKM120 might work in our hands. L-363 cells were treated with 0.5μM and 1μM of BKM120 or GDC0032 for 3 hours. Then, cells were lysed and analyzed by Western blot. C) L-363 was left untreated or treated with indicated concentrations of BKM120 for 96 hours. Next, cell growth was measured by Cell Titer Glo according to the manufacturer’s instructions.
(EPS)

S4 Fig Combined blockage of mTOR and MEK pathways reduces cell growth synergistically at different time points. A) CHP-212 cells were treated with MEK162 or Everolimus or combinations thereof as indicated for 48h. Then, cell growth was assessed by Cell titer Glo. B) Same as C) but the readout was done after 72h. C) Same as B) but the readout was done after 96h. Combination index (CI) values with CalcuSyn Software (Biosoft).
(EPS)
