# Correction: Targeting the mTOR Complex by Everolimus in NRAS Mutant Neuroblastoma

**DOI:** 10.1371/journal.pone.0170851

**Published:** 2017-01-20

**Authors:** Michael K. Kiessling, Alessandra Curioni-Fontecedro, Panagiotis Samaras, Silvia Lang, Michael Scharl, Adriano Aguzzi, Derek A. Oldridge, John M. Maris, Gerhard Rogler

The seventh author’s name is spelled incorrectly. The correct name is: Derek A. Oldridge.
